# Identifying patients at risk of nursing home admission: The Leeds Elderly Assessment Dependency Screening tool (LEADS)

**DOI:** 10.1186/1472-6963-6-31

**Published:** 2006-03-13

**Authors:** Anita Slade, Jon Fear, Alan Tennant

**Affiliations:** 1 School of Allied Health Professions, Leeds Metropolitan University, Leeds, UK; 2 Department of Public Health, Leeds West Primary Care Trust, Leeds, UK; 3 Academic Unit of Musculoskeletal and Rehabilitation Medicine, University of Leeds, Leeds, UK

## Abstract

**Background:**

Discharge from hospital to a nursing home represents a major event in the life of an older person and should only follow a comprehensive functional and medical assessment. A previous study identified 3 dependency scales able to discriminate across outcomes for older people admitted to an acute setting. We wished to determine if a single dependency scale derived from the 3 scales could be created. In addition could this new scale with other predictors be used as a comprehensive tool to identify patients at risk of nursing home admission.

**Methods:**

Items from the 3 scales were combined and analysed using Rasch Analysis. Sensitivity and specificity analysis and ROC curves were applied to identify the most appropriate cut score. Binary logistic regression using this cut-off, and other predictive variables, were used to create a predictive algorithm score. Sensitivity, specificity and likelihood ratio scores of the algorithm scores were used to identify the best predictive score for risk of nursing home placement.

**Results:**

A 17-item (LEADS) scale was derived, which together with four other indicators, had a sensitivity of 88% for patients at risk of nursing home placement, and a specificity of 85% for not needing a nursing home placement, within 2 weeks of admission.

**Conclusion:**

A combined short 17-item scale of dependency plus other predictive variables can assess the risk of nursing home placement for older people in an acute care setting within 2 weeks of admission. This gives an opportunity for either early discharge planning, or therapeutic intervention to offset the risk of placement.

## Background

The National Service Framework for older people within the UK highlighted the need for a single assessment process to determine the most appropriate setting for ongoing care [1]. To date, professionals are often faced with a large assortment of scales to choose from, identifying measures to aid them in this decision was seen as a priority. As the setting of care is largely determined by the extent of dependency and, for example nursing needs, then clearly measures of dependency will be important in this process. A previous study examined the use of 7 outcome scales and other predictive factors (e.g. presence of pressure sores) in order to identify which scales were predictive of outcome when the patient had recently entered an acute hospital setting[2]. Out of the 7 scales examined only four scales, The Modified Barthel Index (MBI) the Abbreviated Mental Test (AMTS), the Northwick Park Dependency Scale (NPDS), and the Caregiver Strain Index (CSI) were found to discriminate across outcomes as defined by placement, for example to a nursing home [3-7].

Mindful of the fact that using several different scales can be time consuming for staff and stressful for the patient we wished to determine whether it was possible to create a screening tool to identify dependency consistent with a nursing home placement from three of these health status scales (MBI, AMTS, NPDS). The CSI was not included as not all patients have a carer, and the three other scales were, in theory, measuring an underlying construct of dependency. If combining some of the original items, together with other key predictive variables into a new scale, could create an algorithm to act as a screen for such risk, this may provide an economical way of assessing the likely need for a nursing home admission.

## Methods

### Objective

To assess if a screening tool (the Leeds Elderly Assessment Dependency Scale (LEADS) could be developed from three previously identified scales which discriminate for nursing home placement [2]. To test if this scale, along with other key predictive variables (identified in previous study), would be sensitive and specific to predicting the need for a nursing home placement within two weeks of admission to acute wards for the Care of the Elderly.

### Participants

Patients were recruited on admission to the Care of the Elderly wards in a multi-site acute hospital trust. A random sample of every fourth patient admitted to three such wards, together with all patients requiring a comprehensive assessment were included in the study. Full details of recruitment and patient characteristics are given elsewhere [2].

### Outcome

Outcome was defined in terms of discharge destination i.e. whether the person was discharged to home, home with family/carer, sheltered housing, residential or nursing home care (in the initial study). In this paper we are primarily concerned with differentiating between nursing home placement and the 'other' placements, as nursing home placement has considerable impact on patients, their families, and on service provision.

### Statistical Methods

**Figure 1:**
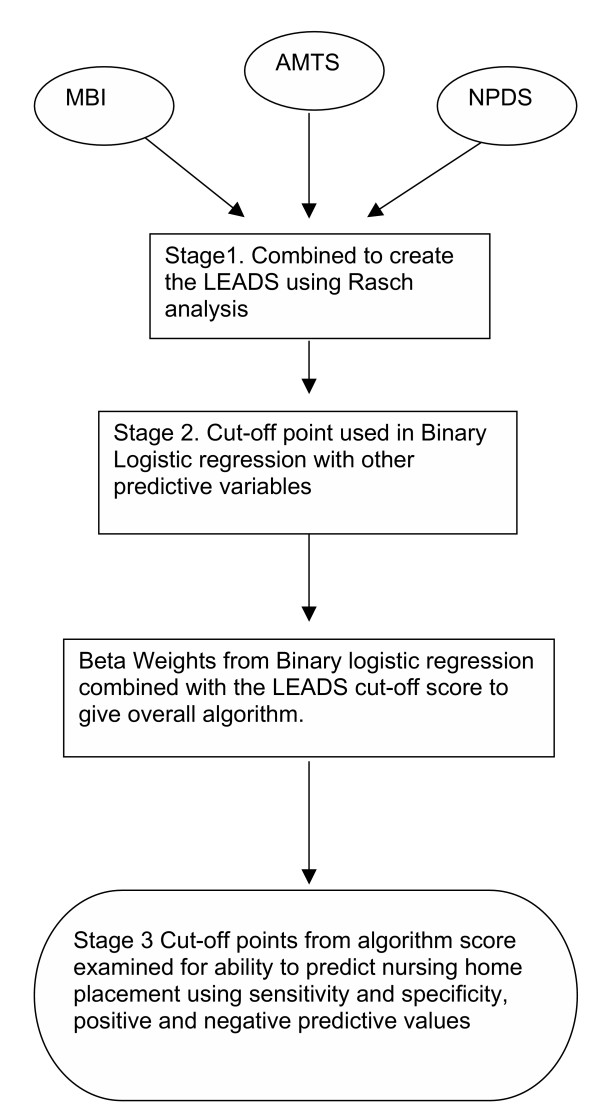
Statistical Methodology Analysis Pathway.

A three stage approach was used to develop the screening tool. (Figure 1)

#### Stage 1: Rasch analysis, developing the LEADS

The three scales were combined and the development of a single shorter scale was explored using Rasch analysis [8]. The Rasch model is the current standard for the development of unidimensional scales (e.g. of impairment or dependency) delivering metric quality outcomes in health care [9]. Briefly, data collected from scales completed by clinical staff, which are intended to be summated into an overall score are tested against the expectations of the Rasch measurement model. The model defines how responses to items should be if measurement (at the metric level) is to be achieved. This was considered the most appropriate model for identifying items that could measure the underlying latent trait (level of disability) into a short assessment tool as it is the only measurement model delivering a metric transformation of ordinal scale [10,11]. A previous study constructed short form scales using this method without any loss of validity, and found it to be a better method for item reduction than more classical forms of item reduction e.g. principal components analysis [7]. Wolfe in his paper discussed different mechanisms for combining scales [10]. The method chosen for the current analysis was the common person equating method. That is the same patient is assessed, at the same time, and assigned values (by clinicians) to each item on the three scales. In looking for potential items to discard, items that were identified as redundant by the Rasch analysis (that is they had high negative residuals, equating to high item-total correlations in classical analysis), were discarded. Also, items that failed to follow the expected probabilistic relationship of a valid scale (misfitting items), were also discarded. Finally, items that showed bias for external factors such as age and gender (Differential Item Functioning were also removed) [13].

The most appropriate cut off point was determined by the sensitivity, specificity, likelihood ratio and ROC curves [14]. Deeks and Altman suggest that likelihood ratios have more powerful properties making them more appropriate in clinical use than sensitivity and specificity alone [15]. Likelihood ratios is the ratio of the probability of finding people who will need nursing home placement to the probability of predicting those patients who will not be at risk of nursing home placement using a defined cut-off score. For comparison, ROC curves are also presented [16].

#### Stage 2: Binary Logistic Regression

Following the construction of the LEADS, a binary logistic regression analysis was used to identify the screening cut-off score derived from the LEADS in combination with the other variables [17]. As the numbers going into a nursing home were expected to be relatively small, it was expected that this would cause some problems in interpretation. King and Zeng identified the difficulties in analysing rare event analysis in logistic regression and advocate that all the rare event cases are used and random sample of the remainder [18]. Consequently this strategy was adopted here, with repeated random samples (in the event of 50 cases) taken from the remainder (the non-nursing home cases) in a one-to -two ratio of those placed in nursing home and non-nursing home cases. The exp (B) values from the binary logistic regression for the indicator variables, together with the LEADS cut-off score were used in an algorithm to create an overall algorithm score for the risk of nursing home placement [19]. (See Appendix 1)

#### Stage 3: Sensitivity, specificity and likelihood ratios

The predictive score for nursing home, identified by the algorithm was then examined for sensitivity and specificity to identify the best predictive cut-off score for the total algorithm. The aim was to maximise sensitivity, and minimise false positives and again, for comparison, ROC curves are presented [20].

### Ethics and Consent

Ethical committee approval was obtained from the Leeds Teaching Hospital NHS Trust. Patients were asked to sign a written consent form. If they were unable to give consent due to cognitive, visual or communication problems, a relative or carer was asked to consent on their behalf.

### Software

Statistical analysis was undertaken with SPSS version 11.5 and RUMM 2020 [21,22].

## Results

Five hundred and forty nine patients were recruited into the full study, of whom 258 were assessed on each of the three scales to be analysed as potential contributions to the screening tool, and discharged to various destinations [2]. The mean age of these 258 patients was 83.8 years (SD 5.5) and their mean length of stay was 31.2 days (SD 31.6). Seven out of ten (70.2%) were female.

### Stage 1: Reducing the item set and producing a single scale: using Rasch Analysis

Items from the 3 scales were merged and examined with the Rasch model. Items that showed misfit or interdependency were discarded, as were those showing bias for age and gender. Reducing the item set in this way gave a 17-item scale with a unidimensional construct of dependency that incorporated both cognitive and functional ability items. The new scale, called the Leeds Elderly Assessment Dependency Screening Tool (LEADS) included 7 items from the MBI, 3 from the AMTS and 7 from the NPDS (Table 1). The overall fit to the Rasch model of this common 17-item scale was good, with Item Fit of -0.414 (SD 1.013), Person Fit of -0.241 (SD 0.559) and Item-trait interaction of (0.004) (Bonferroni correction significance used 0.0006) [23].

Enteral feeding (NPDS 8.3) was the item with the highest negative location (-5.799 logits). This means that the majority of people did not require enteral feeding. In contrast the stairs item (MBI 3) had the highest positive location (+5.031 logits), suggesting that the majority of people found stairs difficult, and independence in this activity was difficult for this group to achieve (Table 1).

**Figure 2:**
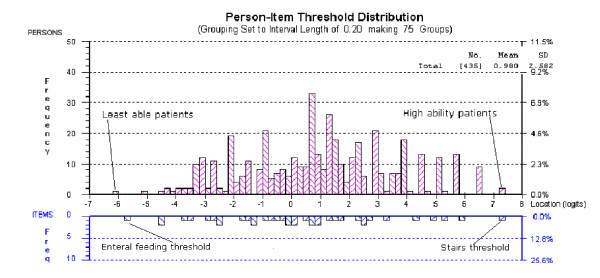
Person item threshold map showing distribution of people and items on the LEADS.

Distribution of people and items for the 17-item scale is good, as shown by the person separation index of 0.944, this indicates that the scale is able to discriminate between several different groups of patients [24]. A Principal components analysis of the residuals showed, with a non-significant Bartlett's test, that no patterns remained in the residuals, thus supporting the unidimensionality of the 17-item scale. The distribution of people and item thresholds can be seen in Figure 2. Each person is shown located on the logit metric scale and thus the raw score from the LEADS can be transformed into a linear metric number [25]. The metric logit location scores from the LEADS (ranging from approximately -6 to + 7 logits) were converted to the range of 0 – 39 (i.e. the same as the original raw score from the contributing items, to facilitate ease of interpretation).

**Figure 3:**
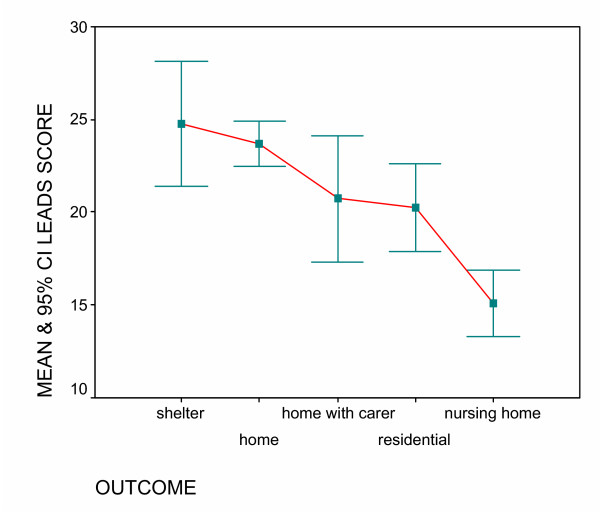
Mean and CI of LEADS score by outcome.

**Figure 4:**
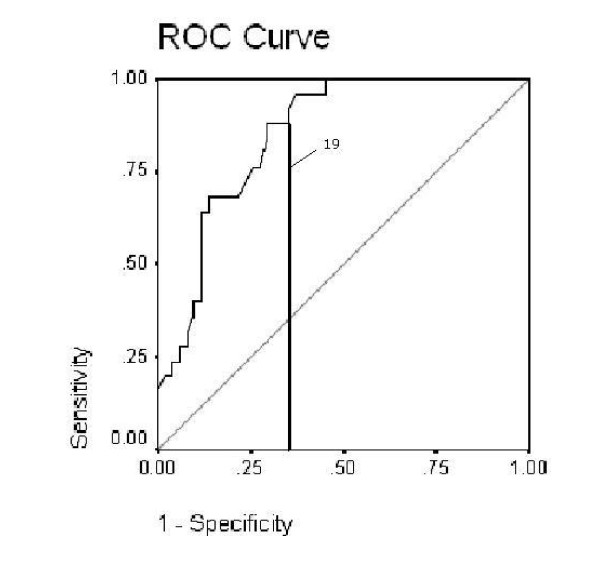
ROC curve of the LEADS showing cut score.

The mean score for those discharged to a nursing home was found to be 15.07 (SD 4.33), while those discharged home without a carer was 23.69 (SD 8.17) (Table 2). There is a significant difference of the LEADS score by discharge destination (Kruskal-Wallis sig. <0.001) (Data was found to be bimodal) and this is shown in Figure 3. As scores for destinations other than nursing home were similar (and had overlapping confidence intervals) we grouped all these destinations into an overall 'other' category. The ROC curve and sensitivity and specificity of different cut points identified the score of 19 as being able to maximise sensitivity (88%) and minimise specificity (61%). With an area under of the curve of 0.81 (SE .036) sig 0.000 (CI 0.738–0.881) this shows that the LEADS score has good predictability as a test for nursing home admission. (Figure 4)

### Stage 2: Predicting the need for nursing home placement: Binary logistic regression

Using the nursing home and the combined 'other' groups as a dependent variable, a binary logistic regression was used to identify predictors for patients at risk of a nursing home placement. There were a disproportionate number of cases between these groups (233:25) and thus, five random samples were selected from the 'other' group and added to the nursing home group to create repeated samples for analysis. The results were consistent for all samples and thus the results presented are the sample that gave the best predictive model.

The best model to predict nursing home placement included the LEADS (with its cut score at 19) together with respite care on admission, communication difficulties on admission, family or patient wishes for placement, and pressure sores (grade 1 or above). The -2 log likelihood statistic (27.227) is analogous to the error sum of squares in multiple regression and is an indicator to how much unexplained information there is after the model is fitted (Table 3). The main model (including all of the variables) is explaining approximately 75% of the variance in predicting nursing home need (chi square (<0.01)); this is significantly better than the best model from the five samples which does not use these predictors.

The coefficients derived from this analysis were then used as the basis of the full algorithm for predicting nursing home placement (Table 4). Exp (B) indicates the change in odds resulting from a unit change in the predictor. For example having a score of less than 19 in the LEADS increases the odds of needing a nursing home admission sixty-five times. Each of these values were included in the algorithm (see Appendix 1).

### Stage 3: Sensitivity, specificity of the final algorithm

**Figure 5:**
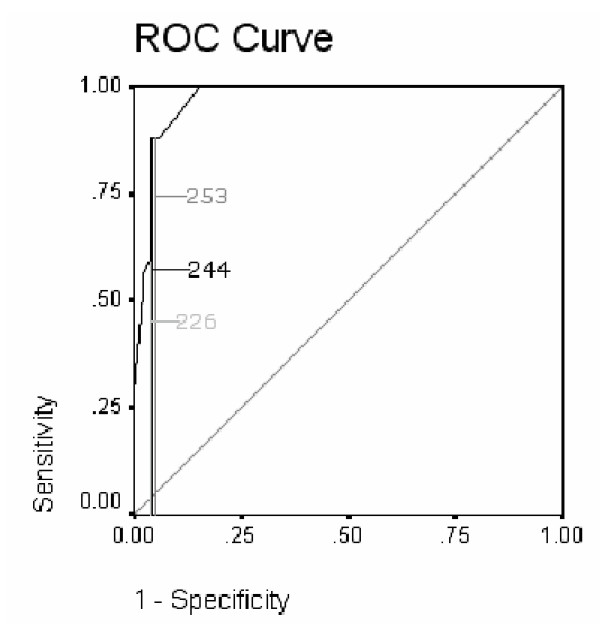
ROC curve showing sensitivity and 1- specificity of cut-points algorithm in sample.

Initially the random sample, which gave the best result for the LEADS was used to determine the best cut score for the full algorithm score based upon the exponentiated values of the logistic regression, and ranging from 0 to 358. This gave a cut score of 244, with sensitivity of 0.88 and specificity of 0.96, and a positive predictive value of 0.92. The likelihood ratio (22.44) was maximised at this value. In addition ROC Curves (figure 5) showed that the area under the curve was 0.972 (SE .016) and this was significant p < 0.000 CI 0.940 to 1.004.

The algorithm was then re-tested on the original full data set of those discharged to home or sheltered accommodation, to residential care, or nursing home care., Thus 258 patients were used to test its sensitivity, specificity and predictive power. Examination of the ROC curve identified 244 as the score that maximised sensitivity (88%) and minimised specificity (85%) (Table 5). The area under the curve for the algorithm score was 0.921 (SE 0.019, sig. 0.000, CI 0.883 to 0.959). Therefore the algorithm score is an excellent predictive test and with a likelihood ratio of 6.04 this indicates that given an algorithm score of less than 244 there is a moderate increase in the likelihood that patients will require nursing home admission. It identified 34 patients needing nursing home that subsequently went elsewhere and identified 3 as 'other' who eventually went to a nursing home. Of the 34 who were predicted as needing nursing home and went elsewhere, 22 (64.7%) returned home, 3 (8.8%) returned home with a carer, 7 (20.6%) went to residential accommodation and 2 (5.9%) went to sheltered housing.

## Appendix 1 -  Algorithm

The algorithm for predicting nursing home placement using SPSS based on minimum Exp B

Compute predscrn = 358

if (LEADS score le 19) predscrn = predscrn -65

if (familypat wish = 1(no)) predscrn = predscrn -34

if (communication difficuties = 1 (yes)) predscrn = predscrn -112

if (grade 1+ pressure sore = 1 (yes)) predscrn = predscrn -18

if (respite care on admission = 1 (yes)) predscrn = prdescrn -128

Excel spreadsheet with the algorithm and scoring for the LEADS is available from:

a.slade@leedsmet.ac.uk or a.tennant@compuserve.com

## Discussion

Currently, people are faced with a bewildering variety of potential measures for use in assessing outcome in an acute elderly setting. Having previously identified three scales that discriminated between people going to a nursing home as against other outcomes, we have now shown that it is possible, through Rasch analysis, to extract items that work well together and measure the underlying dependency trait. Clinicians may still wish to use the original scales for clinical purposes but in terms of measurement, the 17-item LEADS scale and associated algorithm has been shown to be a powerful tool in predicting patients at risk of nursing home placement and those likely to go to other types of care or home.

The false positive rate in the final analysis may be viewed as a major weakness in the approach. Some patients improved such that they could go home, or into other institutional settings. This is a valid comment and the majority of mismatch between the indicative and final placement was for those patients who went home. It is important to remember this data was collected within two weeks of admission. Thus the algorithm, as well as providing a common equitable means of assessment, can act as an early warning system for risk of institutional care. Early identification of those patients at risk enables interventions to be instigated early on in their admission, potentially reducing the risk of nursing home placement. Given the parsimony of the scale there is nothing to prevent repeated measurements during the patients stay in hospital, so providing a monitoring system for the continuing risk of institutional placement.

There are a number of weaknesses to the study. The low number of patients subsequently entering a nursing home was always going to be a cause of concern. However, we accommodated this, as best as possible, by sampling from the other group and comparing the results. The assessment was also only undertaken once within two weeks of admission. Additional work needs to be carried out using repeated assessments and to look at the changes in sensitivity and specificity over time in order too determine if there is an optimum time to maximise these parameters. Due to the low numbers of those entering a nursing home, we had to use this group in the development of the algorithm cut point, as well as its validation. This is likely to overestimate its predictive value, although we did try to offset this as far as possible by validating the algorithm on the full data set, rather than the developmental sample. Finally, as with all models developed on a particular set of data, these results need replicating on other elderly acute samples to support conclusions about the predictive validity of the screening tool.

## Conclusion

Using selective items from three separate scales, previously shown to be discriminative for nursing home placement, together with other key indicators, enables those working in an acute setting, within two weeks of admission, to identify 85 % of patients at risk of needing nursing home placement. The resulting LEADS scale and four indicator variables can easily be administered by any health care professional and the risk algorithm lends itself to a simple spreadsheet calculation.

## Abbreviations

## Competing interests

The author(s) declare that they have no competing interests.

## Authors' contributions

AS, AT and JF conceived the study question and design.

AS was the principal data analyst.

AS wrote the provisional drafts of the manuscript with AT and JF reviewing the manuscripts.

AS was responsible for data collection and fieldwork.

All authors contributed to the critical evaluation of the methods, analysis and writing.

AT is the guarantor of the study.

All authors read the final manuscript.

## Pre-publication history

The pre-publication history for this paper can be accessed here:



## Figures and Tables

**Figure 6:**
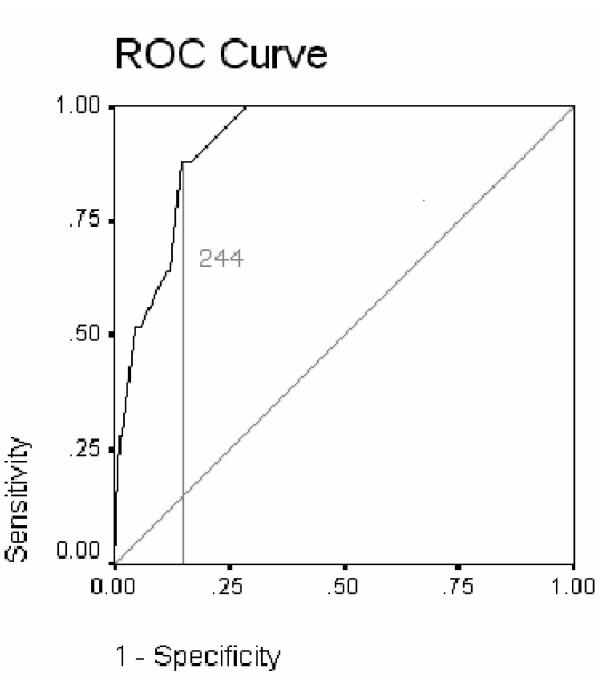
ROC curve showing sensitivity and 1-specificity of algorithm score full cohort.

**Table 1:** Items and their location in the LEADS scale

	ORIGINAL SCALE & ITEM NO.	ITEM	LOCATION	SE	RESIDUAL	CHI. SQU	PROB.
1	**MBI 3**	Stairs	5.031	0.190	0.161	4.074	0.130396
2	**MBI 7**	Bathing	3.745	0.180	-1.197	5.015	0.081487
3	***AMT 7***	Know current month	0.852	0.220	2.009	7.429	0.024363
4	**MBI 6**	Grooming	0.837	0.100	-1.887	3.347	0.187607
5	**MBI 2**	Ambulation	0.813	0.130	0.077	3.807	0.149042
6	*NPDS 7*	Dressing	0.327	0.100	-1.591	2.304	0.315995
7	*NPDS 1*	Mobility	0.277	0.120	0.275	0.610	0.736964
8	***AMT 4***	Orientated to place	0.085	0.230	0.840	8.333	0.015506
9	*NPDS 4.1*	Toileting bowels	-0.061	0.120	-0.302	1.552	0.460256
10	*NPDS 2*	Bed transfer	-0.129	0.120	-1.570	7.167	0.027784
11	*NPDS 5*	Wash/groom	-0.224	0.130	-1.178	3.501	0.173663
12	**MBI 9**	Urinary incontinence	-0.281	0.200	0.268	4.049	0.132080
13	**MBI 4**	Feeding	-1.108	0.160	-0.490	3.571	0.167688
14	**MBI 11**	Faecal incontinence	-1.226	0.160	-0.082	2.638	0.267454
15	***AMT 6***	Know DOB	-1.296	0.270	-0.361	0.683	0.710540
16	*NPDS 9*	Skin pressure	-1.844	0.150	0.013	1.182	0.553675
17	*NPDS 8.3*	Enteral Feeding	-5.799	0.520	-0.135	0.546	0.761256

**Table 2:** Mean LEADS score by outcome with confidence intervals

**Outcome **	**N**	**Mean (95% CI)**	**SD**	**SE**	**Min score**	**Max score**
Sheltered housing	24	24.79 (21.43–28.16)	7.972	1.627	13	38
Home without carer	170	23.69 (22.45–24.92)	8.172	.627	5	39
Home with carer	15	20.74 (17.32–24.15)	6.166	1.592	11	35
Residential care	24	20.25 (17.91–22.60)	5.555	1.134	10	35
*Nursing home*	*25*	*15.07 (13.28–16.86)*	*4.332*	*.866*	*7*	*23*
**Total**	**258**	**22.46 (21.49–23.44)**	**7.977**	**.497**	**5**	**39**

**Table 3:** Binary logistic regression fit statistics

	-2 LOG LIKELIHOOD	NAGELKERKE R SQUARE	COX & SNELL R SQUARE	% CORRECTLY IDENTIFIED
Leads	71.113	.282	.392	88
All variables including LEADS cut-off 19	27.277	.829	.597	92.0

**Table 4:** Minimum Exp B Variables from logistic regression and range over 5 samples

VARIABLES (RANGE)	B	S.E.	WALD	SIG.	EXP(B)
CUT-OFF 19 LEADS Range	4.181 (2.21–4.18)	1.490 (0.90–1.49)	7.878 (6.06–7.88)	.005 (0.01–0.03)	65.454 (9.10–65.45)
Respite care on admission	4.850 (2.64–4.85)	2.064 (1.29–2.06)	5.522 (4.14–5.52)	0.019 (0.01–0.064)	127.793 (14.07–127.79)
Communication difficulties on admission	4.722 (2.64–4.72)	1.688 (0.76–1.69)	7.823 (7.82–12.57)	0.005 (0.00–0.01)	112.425 (10.66–112.42)
Family/patient expressed wish for placement	3.54 (2.43–3.61)	1.636 (1.08–1.64)	4.669 (4.67–5.97)	.031 (0.01–0.03)	34.269
Grade 1 plus pressure sore	2.872 (1.13–2.87)	1.291 (0.76–1.29)	4.952 (2.22–4.95)	.026 (0.03–0.14)	17.678 (3.10–17.68)
Constant	-8.365	2.471	11.462	.001	.000

**Table 5:** Specificity and sensitivity of the cut scores from the LEADS algorithm full cohort

CUT-OFF SCORES	SENSITIVITY	SPECIFICITY	LR	PPV	NPV
236	0.64	0.88	5.33	0.364	0.958
**244**	**0.88**	**0.85**	**6.03**	**0.393**	**0.985**
253	0.88	0.83	5.26	0.361	0.985

Co-ordinates of the ROC curve identify cut-off values as an average of two consecutive ordered test values

Sensitivity is the proportion of patients that are correctly identified as needing nursing home.

Specificity is the proportion of people that are correctly identified as not needing nursing home.

LR+ = Likelihood ratio, how many more times likely patients will need nursing home with this cut-off score than those who do not.

Positive predictive value is the proportion of people who had a positive test for nursing home who did need a nursing home admission.

Negative predictive value is the proportion of people who had a negative test result and didn't need a nursing home admission
